# Facile synthesis, physiochemical characterization and bio evaluation of sulfadimidine capped cobalt nanoparticles

**DOI:** 10.1016/j.sjbs.2021.02.071

**Published:** 2021-03-04

**Authors:** U. Nagababu, J.V. Shanmukha Kumar, Mohammed Rafi Shaik, Mohammed A.F. Sharaf

**Affiliations:** a Department of Chemistry, Koneru Lakshmaiah Education Foundation, Vaddeswaram, Guntur, Andhra Pradesh 522502, India; b Department of Chemistry, College of Science, King Saud University, P.O. Box 2455, Riyadh 11451, Saudi Arabia; c Department of Industrial Engineering, College of Engineering, King Saud University, P.O. Box 800, Riyadh 11421, Saudi Arabia

**Keywords:** Cobalt nanoparticles, Sulfadimidine, Chemical reduction, Biological activity, Capping agent

## Abstract

Due to their less expensive, environment friendly nature, and their natural abundance of cobalt have attained more significant attention for the synthesis of cobalt nanoparticles. In the present study, we report the facile synthesis of cobalt nanoparticles using a straight forward chemical reduction approach of cobalt chloride with sodium borohydride and capping of sulfadimidine. sulfadimidine has strong capping eligibility on the surface of nanoparticles due to its chemical stability and is an applicable as stabilizer due to the existence of an amine bond. The as-synthesized sulfadimidine stabilized cobalt nanoparticles (Co-SD NPs) were characterized by using various spectroscopic and microscopic analysis like UV–Visible spectroscopy (UV–Vis), X-ray powder diffraction (XRD), scanning electron microscopy (SEM), High-Resolution Transmission electron microscopy (HR-TEM), and Fourier-transform infrared spectroscopy (FT-IR). The XRD analysis exhibited the triclinic crystal structure of the as-synthesized cobalt nanoparticles and FT-IR analysis confirmed the capping of sulfadimidine via monodentate interaction. The HR-TEM analysis displayed the size of the cobalt nanoparticles approximately 3–5 nm. The antibacterial properties of the sulfadimidine stabilized cobalt nanoparticles (Co-SD NPs) were tested against various bacterial strains such as *Klebsiella pneumonia (KP), Escherichia coli (EC) and Pseudomonas syringae (PS)* by using agar disc diffusion approach. The results of sulfadimidine capped cobalt nanoparticles displayed the enhanced biological properties against the tested gram-negative bacteria.

## 1 Introduction

**Scheme 1:**
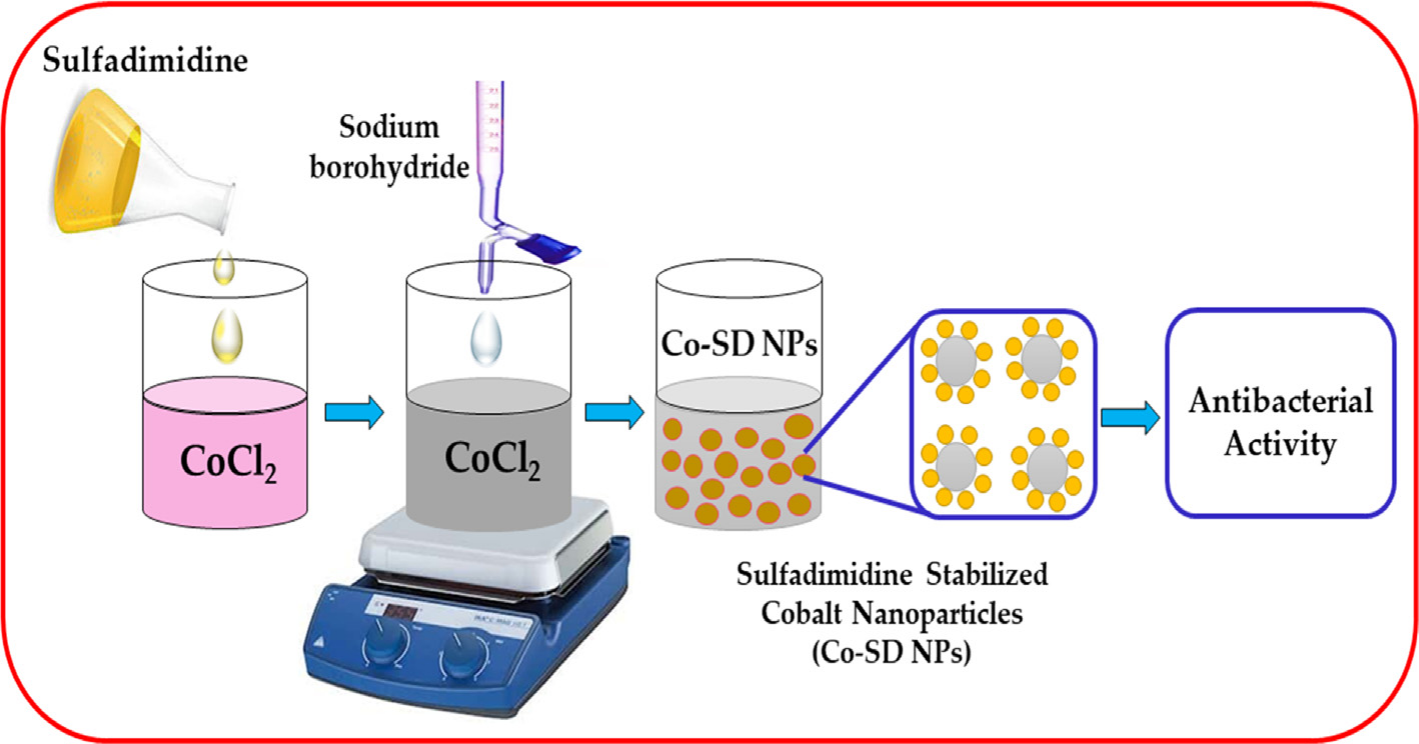
Schematic Representation of as-synthesized sulfadimidine capped cobalt nanoparticles and their evaluation of antibacterial activity.

Nanoscience has attained more consideration owed to their wide range of applications (Khan et al., 2018, Sanchez and Sobolev, 2010). Generally, nanomaterials which comprise at least a dimension less than 100 nm (Khan et al., 2018). They display versatile properties like chemical, physical and biological properties from their different from bulk materials. Dependent on the inclusive structure these nanomaterials can be different dimensional such as zero, one, two and three (0D, 1D, 2D and 3D) (Rafiei-Sarmazdeh et al., 2019). Usually nanomaterials are generally categorized into different types based on their size, structure and chemical properties (Jiang et al., 2019). Metal nanoparticles, a novel vocabulary has been initiated in the nanotechnology field in current years. The noble metal nanoparticles like platinum, silver, palladium, and gold having advantageous possessions on health are exploited for the fabrication of nanomaterials and labelled as metal nanomaterials (Czubacka and Czerczak, 2019, Das et al., 2019, Pedone et al., 2017, Yaqoob et al., 2020, Zhang et al., 2020). Currently investigators and scientists are concentrating on metal nanomaterials and nanostructures preparations because of their noticeable properties that are beneficial for polymer composite, coatings, catalysis, sensor, and medical etc., (Maduraiveeran and Jin, 2017, Pirzada and Altintas, 2019, Shah and Li, 2019) (see Scheme 1).

The fabrication of nanomaterials with controlled size and shape has boosted nanotechnology into one of current most significant filed in the research (Dahoumane et al., 2017, Khor et al., 2019). Possible upcoming development entails the capability to synthesize nanomaterials in a controlled and reproducible method. Presently, there is a collective attention to fabricate metallic nanoparticles not only for the development of synthetic progress, but also for the estimation of their properties such as catalytic, sensors and biological (Falahati et al., 2020). Presently more consideration has been paid to the metal nanoparticles synthesis and characterizations (Jamkhande et al., 2019). The chemical approach has been recognized to develop desirable structure and size of the metal nanoparticles in a facile approach. Control over morphology and size is attained by a well considerate of fundamental measures (Rodrigues et al., 2019), the mechanism by transformation of the precursor, stabilizing agent, and solvent in the method and its association with development and rate of nucleation (Esther and Sridevi, 2017). Diverse physicochemical reduction approaches like photochemical, chemical, electrochemical are usually active for the development and stabilization of metal nanoparticles. The choice of synthesis approach of metal nanoparticle is correspondingly significant for the reason that during nanoparticle production progressions for instance kinetics of interface of metal ions with reducing agent, adsorption procedure of stabilizing agent with metal nanomaterials and various investigational methods generates strong impact on its physicochemical properties, size and structure (Akter et al., 2018).

Numerous metal particles existing in the products for instance medicines, tooth paste, cosmetic, detergents, and pharmaceutical drugs are straight approaching in interaction with human. Metallic nanoparticles functionality and activity based on the microstructure, size, morphology and microstructure. From microstructure perspective, Cobalt has been examined widely owing to circumstance that it displays flexible crystal structures (Ansari et al., 2017, Dey and Dhal, 2019, Fadheela et al., 2020). Moreover, cobalt nanoparticles generally enticing vast attention owed to their exceptional structure and size dependent properties and potential uses (Khan et al., 2015).

Furthermore, the outstanding properties catalytic have been stated for diverse structures of cobalt nanoparticles (Kamal et al., 2019). Therefore, efforts have been conducted to attain various structures and sizes such as spheres, sheets, snowflakes or cauliflower like particles, and flakes etc. A wide range of development actions, such as solvothermal, hydrothermal, evaporation of metals, microemulsion, sonification, reduction of cobalt salt, electrochemical generation in the existence of surfactants, were implemented to produce cobalt nanoparticles with variable structure and size (Zola et al., 2014). Usually, Cobalt nanoparticles hold magnificent electrical, chemical and magnetic properties that significant attention in diverse fields, together with magnetic sensors, composites, reminiscences, and fluids etc., (Imadadulla et al., 2018, Michalek et al., 2008).

Cobalt nanoparticles were developed using a facile chemical reduction approach because of its comparative merits like inexpensive, ecofriendly, and sustained aqueous phase etc. Furthermore, cobalt nanoparticles process without having higher temperatures while retaining their biocompatibility, consideration is focused in the direction of assessing the biocompatibility of cobalt nanoparticles. Consequently, the objective of present work cobalt nanoparticles (Co-SD NPs) synthesized by using chemical approach using sodium borohydride in the presence of sulfadimidine has been used as a stabilizing or capping agent, as of it delivers the steric stabilization of the as-synthesized cobalt nanoparticles (Co-SD NPs) contrary to the van der Waals interactions and in this manner avoids their agglomeration. Furthermore, this method boundaries the development of the nanoparticles and stops the Wilhelm Ostwald ripening technique since its layer of the surface performances as a barrier to the mass transfer. By using numerous techniques as-synthesized cobalt nanoparticles characterized such as HR-TEM, SEM, UV–Vis, XRD, and FT-IR. The biological properties of the sulfadimidine capped cobalt nanoparticles (Co-SD NPs) were studied against various bacterial strains comprising *Klebsiella pneumonia (KP), Escherichia coli (EC), and Pseudomonas syringae (PS)*. The cobalt nanoparticles disclosed substantial activity against the investigated bacterial strains.

## 2 Materials and methods

### 2.1 Materials

The chemicals were procured from Sigma-Aldrich (USA), Cobalt(II) chloride (>99%), Sodium borohydride and dimethyl sulfoxide (>99.9%). sulfadimidine was acquired from AU-Organic & Foods, Drugs and Water Lab (India). All materials were used as received.

### 2.2 Preparation of sulfadimidine capped cobalt nanoparticles (Co-SD NPs)

In a characteristic synthesis approach, 0.1 M CoCl_2_ was dissolved in to 100 ml distilled water. In the next step, 50 ml of 0.1 M sulfadimidine drug was mixed to the solution as stabilizer with stirring. Further, a solution of 20 ml of sodium borohydride was added to the CoCl_2_ reaction mixture, and the reaction was persistently stirred for about 30 min at room temperature. The Cobalt colloid was attained which was understood by the change of the color during the reaction gradually, initially from pink to colorless and finally to gray color. The attained product was washed and separated using centrifugation and dried at 60 °C for 12 h.

### 2.3 Characterization

As-synthesized cobalt nanoparticles (Co-SD NPs) crystalline phase was studied by using x-ray powder diffraction (XRD) measured on PANalytical's XPERT-PRO using Cu K_α_ (λ = 1.54059 Å). The spectral analysis of the cobalt nanoparticles was conducted using UV–Visible (Shimadzu UV-3100 spectrophotometer). The HR-TEM analysis carried out with Hitachi H-8100 IV at 200 kV. The SEM examination conducted using JED-2200, JEOL, Japan. The FT-IR analysis of the cobalt nanoparticles carried out using Bruker IFS 66 v/S spectrometer, Germany.

### 2.4 Preparation of bacterial inoculum

The selected bacteria were injected into Muller Hinton broth (MH broth) and incubated at 35–37 °C for 24 h. The resulting suspensions turbidity was dilute with MH broth to attain a transmittance. That attained percentage was analogous to 1 McFarland turbidity standard. This turbidity is comparable to around 3 × 10^8^ CFU/ml. The spectrophotometer (Spectronic 20, Bausch & Lomb®) was castoff to regulate the working suspensions transmittance. This suspension cast-off as inoculum.

### 2.5 Agar well diffusion assay

Perez *et al*., procedure was castoff in agar diffusion approach (Perez, 1990). The chosen medium was inoculated with the bacteria placed in MH broth (Osaki et al., 2004). When the agar was coagulated, it was cut with a diameter of 6 mm wells and filled with essential compounds concentration, Ampicillin is employed as standard for positive control while pure Solvents were employed as negative control. Outcomes were attained established on size of the zone of inhibition, adjacent the wells comprising the extract associating with standard and blank. The zone of inhibition was assessed in milliliter using HiMedia zone reader.

### 2.6 Antibacterial activity of sulfadimidine stabilized cobalt nanoparticles (Co-SD NPs)

The antibacterial activity of as-synthesized sulfadimidine stabilized cobalt nanoparticles (Co-SD NPs) was attained using the agar disk diffusion assay approach. Three bacterial strains, American Type Culture Collection namely Klebsiella pneumonia, Escherichia coli and Pseudomonas syringae were accomplished from the Department of Microbiology, Science College, KL University, India. The strains of the bacteria were sub cultured in mmediately fabricated nutrient broth and post observation of growth was constant as per 1 McFarland turbidity standards. The inoculum was kept on nutrient agar plates by the spread disk approach and a sterile tool 6 mm was utilized to bore 4 wells in the prepared plate. Each well, 100 µL of 30 µg/mL each of Co-SD NPs, dimethyl sulfoxide (DMSO) and Ampicillin (50 µg/mL) was added and the subsequent wells continued as the negative control. At 37 ˚C for 24 h cultured plates were incubated to assess the inhibition zone of the examined bacteria. All the examinations were carried out in triplicates to exclude experimental errors.

## 3 Results

### 3.1 UV–Visible analysis

**Fig. 1:**
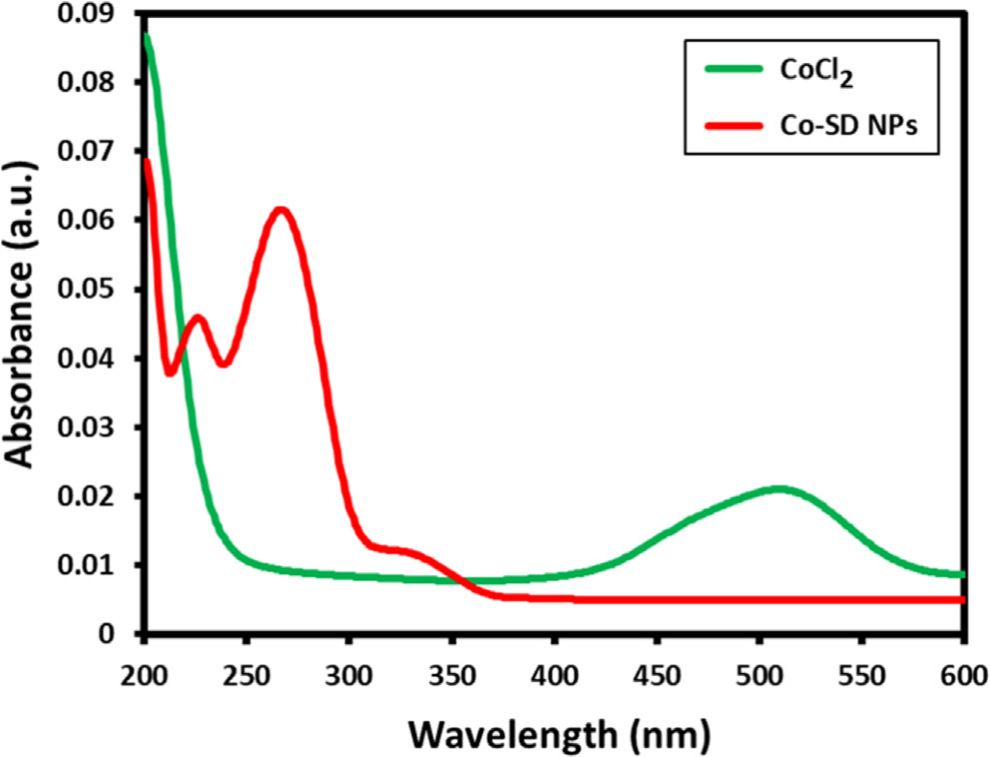
The UV–visible anlysis of Co-SD NPs.

The Co-SD NPs were synthesized by chemical reduction approach using sodium borohydride. The development of sulfadimidine stabilized cobalt nanoparticles Co-SD NPs was characterized by UV–visible spectra, Fig. 1 displays the UV–visible absorption of cobalt chloride and Co-SD NPs. The UV–Vis absorption of cobalt chloride displayed an adsorption band around 460–550 nm, in the case as-synthesized Co-SD NPs The absorption band around 460–550 nm was not noticed and also Co-SD NPs do not have any obvious absorptions in the 460–550 nm region. The attained spectra clearly indicated the formation of Co-SD NPs formation. Additionally, the Co-SD NPs exhibited an additional absorption peaks at 225, 270 and 330 nm (Fig. 1), which is because of the sulfadimidine attached to the as-synthesized Co-SD NPs surfaces as stabilizing agent. These additional peaks confirmed that sulfadimidine existing on the surface of nanoparticles as a stabilizing or capping agent.

### 3.2 XRD analysis

**Fig. 2:**
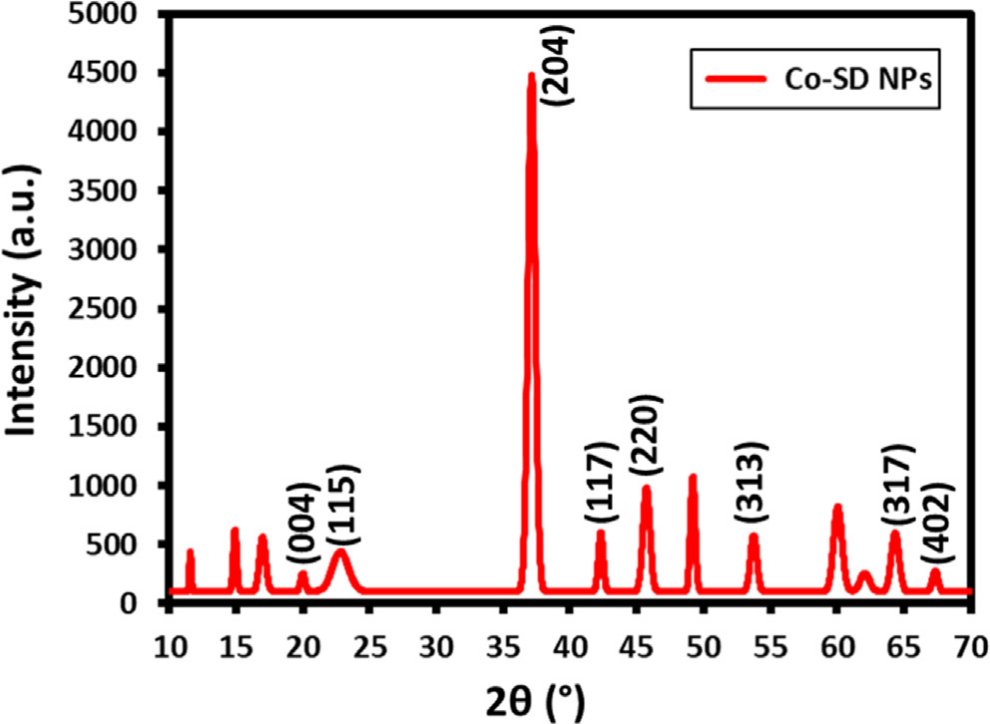
X-ray powder diffraction pattern of Co-SD NPs.

Fig. 2 and Table 1 elucidates the XRD pattern of Co-SD NPs obtained using chemical reduction method. The diffraction peaks appeared at 20.03 (0 0 4), 22.85 (1 1 1), 37.14 (2 0 4), 42.31 (1 1 7), 45.74 (2 2 0), 53.75 (3 1 3), 64.35 (3 1 7) and 67.33 (4 0 2) represents triclinic crystal structure of cobalt nanoparticles (Co-SD NPs). According to Debye–Scherrer formula the crystallite size average attains to be 3.67 nm, nearly in agreement with the particle size obtained from HR-TEM image. The additional diffraction pattern existing in the XRD analysis clearly indicating the existence of sulfadimidine nanoparticles surface.

**Table 1:** XRD analysis and crystallographic data of the Co-SD NPs.

2θ (°)	FWHM (°)	Sin θ (radian)	h,k,l	a (nm)	b (nm)	c (nm)	α (°)	β (°)	γ (°)	Cos θ (radian)	*D* (nm)
20.03	2.24	0.17477	004	0.56	0.56	1.76	90.40482	89.99592	90.54754	0.984767	3.60
22.85	2.22	0.19939	111	0.56	0.56	1.76	90.40482	89.99592	90.54754	0.980188	3.65
37.14	2.28	0.324092	204	0.56	0.56	1.76	90.40482	89.99592	90.54754	0.94794	3.68
42.31	2.29	0.369245	117	0.56	0.56	1.76	90.40482	89.99592	90.54754	0.9326	3.72
45.74	2.34	0.399134	220	0.56	0.56	1.76	90.40482	89.99592	90.54754	0.921398	3.68
53.75	2.39	0.469091	313	0.56	0.56	1.76	90.40482	89.99592	90.54754	0.89198	3.73
64.35	2.53	0.561522	317	0.56	0.56	1.76	90.40482	89.99592	90.54754	0.846446	3.71
67.33	2.56	0.587608	402	0.56	0.56	1.76	90.40482	89.99592	90.54754	0.832269	3.73

The average crystalline size was assessed using the following principle.

$$\frac{1}{d^{2}}=\frac{1}{V^{2}}(S_{11}h^{2}+S_{22}k^{2}+S_{33}l^{2}+2S_{12}hk+2S_{23}kl+2S_{13}hl$$

$$S_{11}=b^{2}c^{2}{Sin}^{2}\alpha$$

$$S_{22}=a^{2}c^{2}{Sin}^{2}\beta$$

$$S_{33}=a^{2}b^{2}{Sin}^{2}\gamma$$

S_12_ = abc^2^ (cos α cos β – cos γ)

S_23_ = a^2^bc (cos β cos γ - cos α)

S_13_ = ab^2^c (cos γ cos α - cos β)

$$V=abc\sqrt{1-{cos}^{2}\alpha -{cos}^{2}\beta -{cos}^{2}\gamma +2cos\alpha cos\beta cos\gamma }$$

$$Debye-Scherrer\;\;\;formula\;\;\;D=\frac{k\lambda }{FWHM\;\;x\;\;cos\;\;\theta }$$

Where, Lattice Constants are “a, b and c”, Interplanar Spacing “d”, Miller Indices are “h,k,l”, and α, β and γ are the axial lengths and angle.

‘D’ is representing Crystal size, wavelength X-ray is “λ”, and ‘k’ denotes the dimensionless factor, the FWHM denotes line broadening at half the maximum intensity, Bragg’s angle ‘θ’.

The assessed crystallite size average approximately to be 3.67 nm, which is almost identical size when relate to the particles size examined by HR-TEM analysis.

### 3.3 SEM analysis

**Fig. 3:**
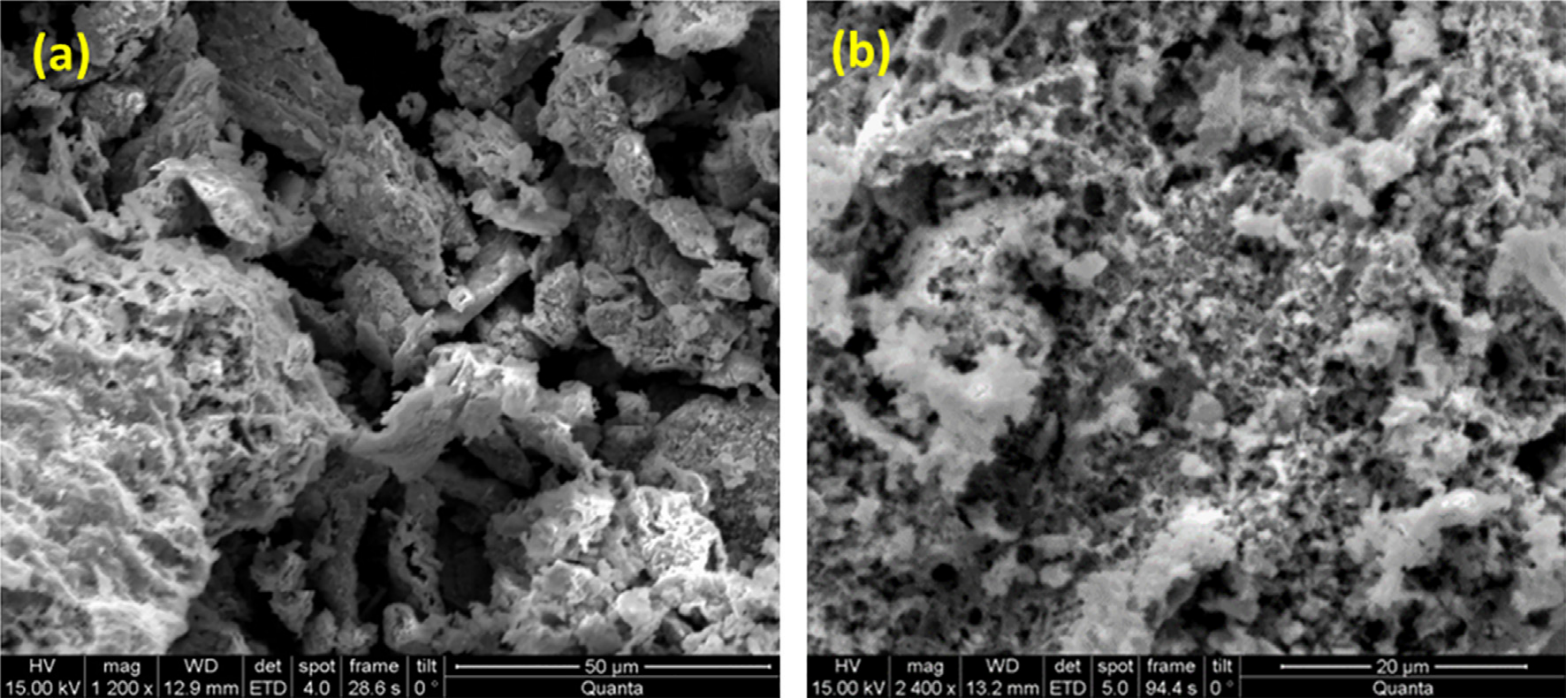
(a) and (b) the scanning electron micrograph of Co-SD NPs with different scale bar.

Fig. 3 elucidates the SEM micrograph of Co-SD NPs. SEM image authorizes the morphology of the as-synthesized cobalt nanoparticles. It is witnessed that the scattered crystalline nature of cobalt nanoparticles without aggregated state. The shape of the Co-SD NPs is not clear from SEM analysis. The SEM results of the cobalt nanoparticles agreement with the XRD results.

### 3.4 HR-TEM analysis

**Fig. 4:**
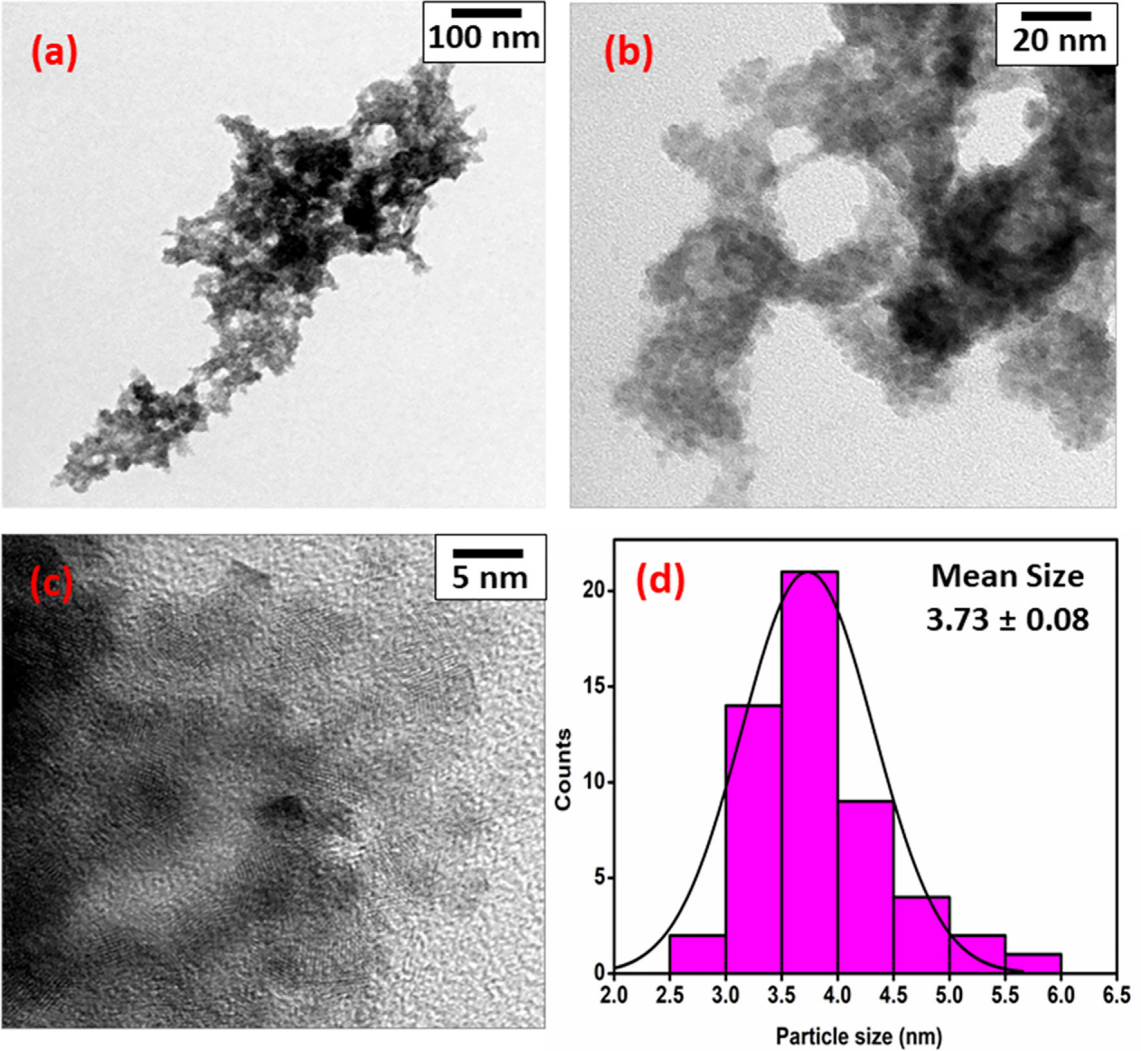
High-Resolution Transmission electron microscope analysis of the Co-SD NPs (a) and (b) overview HR-TEM images of Co-SD NPs; (c) HR-TEM magnified picture, and (d) distribution graph of the particle size Co-SD NPs.

Transmission electron microscopy has been performed to investigate the actual size and shape of the Co-SD NPs. The HR-TEM analysis displayed that the shape of the as-synthesized cobalt nanoparticles is clear from the HR-TEM images in Fig. 4a-4c, that the particles are uniformly and well distributed in nature of average size 3–5 nm. The XRD displays characteristic of triclinic of Co-SD NPs is evidently witnessed. The HR-TEM analysis exhibiting that the structure morphology observed in the HR-TEM image are nano crystalline in nature. It is perceived that the Co-SD NPs are scattered over the surface and no aggregates are noticed. The particles size distribution was calculated by using image J software and average particle it was found to be approximately 3.73 ± 0.08 nm (Fig. 4d).

### 3.5 FT-IR analysis

**Fig. 5:**
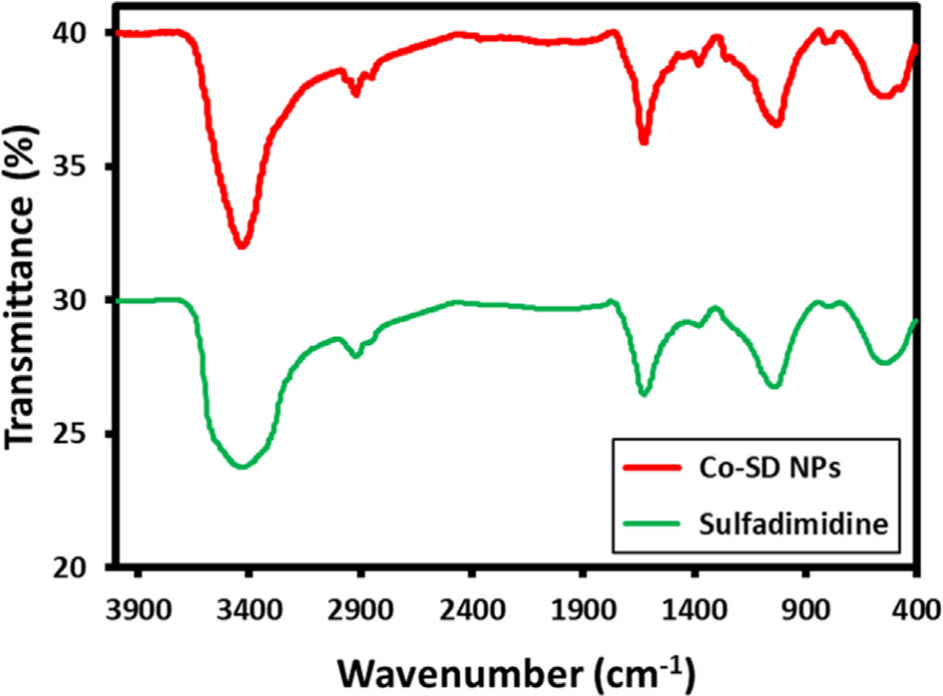
FT-IR spectra of sulfadimidine capped cobalt nanoparticles (Co-SD NPs).

FT-IR analysis was performed to recognize the feasible biomolecules accountable for capping and efficient stabilization of Co-SD NPs synthesized using Sulfadimidine. Fig. 5 exhibits the FT-IR spectrum of sulfadimidine and Co-SD NPs. The FT-IR spectrum of sulfadimidine drug showed prominent absorptions bands at 3367, 3110, 2950, 1626, 1400 and 1056 cm^−1^. In the case of as-synthesized cobalt nanoparticles displayed almost identical peaks in the spectrum such as 3400, 2923, 2865, 1652, 1055, 1031 and 1012 cm^−1^ revealed that sulfadimidine acting as a stabilizing or capping agent. Owing to the presence of sulfadimidine on the nanoparticles surface controls the size and prevents the agglomeration of the nanoparticles.

### 3.6 Antimicrobial activity studies

**Fig. 6:**
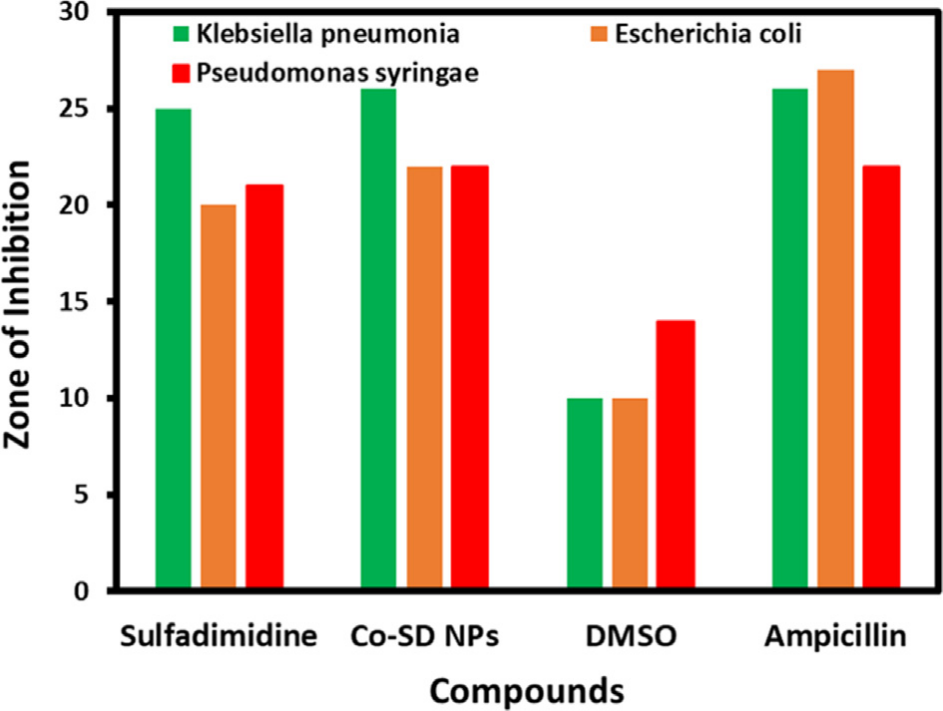
Antibacterial activity of sulfadimidine, DMSO solvent, ampicillin and Co-SD NPs.

The bacteriological studies were carried out using Muller Hilton Broth technique. The as-synthesized cobalt nanoparticles (Co-SD NPs), sulfadimidine, dimethyl sulfoxide (DMSO) solvent and Ampicillin were tested against the gram-negative bacteria (Fig. 6). The Co-SD NPs showed excellent activity against examined bacterial strains. The minimum inhibitory concentration (MIC) of sulfadimidine and Co-SD NPs was estimated as the lowermost concentration at which the bacterial growth was inhibited.

The MIC of pure sulfadimidine for gram-negative bacterial strains was found to be ≥ 4.0 μg/ml. When antibacterial activity of Co-SD NPs was measured using sulfadimidine as capping agents MIC decreased considerably. The MIC of all bacterial species is correspondent to Minimum bactericidal concentration (MBC). The results are shown in Table 2. The sulfadimidine in conjunction with Co-SD NPs is not a capable inhibitor for *E. coli.*

**Table 2:** Zone of Inhibition of the bacterial strains examined against Co-SDNPs and antibiotic Ampicillin.

Compounds	*Klebsiella pneumonia*	*Escherichia coli*	*Pseudomonas syringae*
Sulfadimidine	25	20	21
Co-SD NPs	26	22	22
DMSO	10	10	14
Ampicillin	26	27	22

## 4 Discussion

The chemical preparation of metal nanoparticles can be generally attained by reduction of metallic salt. Zola et al., synthesized cobalt metallic nanoparticles by chemical reduction approach by using sodium borohydride (Zola et al., 2014). In the similar manner Salman et al., successfully synthesized cobalt nanoparticles using chemical reduction approach using hydrazine hydrate and citric acid as a stabilizing agent (Salman et al., 2014). In current study, sulfadimidine capped cobalt nanoparticles successfully synthesized using sulfadimidine and sodium borohydride, sulfadimidine used as a capping or stabilizing agent. Furthermore, the use of sulfadimidine as a stabilizing agent controls the size and also enables the as-synthesized nanoparticles stabilization. This pointedly advances the ability of the nanoparticle’s penetration into the bacteria cell wall and thus helps to attain enriched biological activity of cobalt nanoparticles. The as-synthesized sulfadimidine capped cobalt nanoparticles are characterized by using numerous techniques. The average crystalline size was calculated using XRD analysis was 3.67 nm.

In contrasts among our results in present study and those from other investigators studies are complicated as the procedures cast-off to identify bactericidal activities are diverse from other studies (Esmaeillou et al., 2017). In another examination, Khan et al., developed cobalt oxide nanoparticles by using thermal decomposition approach and tested for their biological activity contrary to several bacterial strains (Khan et al., 2015). In our investigation, the results were showed that, sulfadimidine capped cobalt nanoparticles Co-SD NPs enhanced the antibacterial activities against gram-negative bacteria. It can be impregnable as it is accompanying with the outer membrane of bacteria gram-negative.

In our results, it was detected that the sulfadimidine-bound Co-SD NPs had amplified bactericidal activity related to pure sulfadimidine. Especially the results obtained for *Klebsiella pneumonia (KP)* showed almost equal to that of the ampicillin antibiotic. Whereas, the antibacterial activity on *Escherichia coli* and sulfadimidine are same and comparatively less than reference antibiotic.

Co-SD NPs synthesis might be accomplished with various ligand stabilizers. sulfadimidine has strong capping ability on the Co-SD NPs surface and is an appropriate stabilizer due to the presence of an amine bond was preferred owed to its chemical stability (Kora and Rastogi, 2013). The consequences exhibited that *in vitro* antibacterial activity against gram-negative bacteria by sulfadimidine capped Co-SD NPs onto the surface. Moreover, the activity was showed nearly equal on *Klebsiella pneumoniae* when compared with ampicillin antibiotic. The Co-SD NPs results displayed against the tested gram-negative bacteria with zone of inhibition of *Klebsiella pneumonia* 26 mm*, Escherichia coli* 22 mm*, and Pseudomonas syringae* 22 mm. The as-prepared Co-SD NPs were found to be active against the examined bacteria and signifying their potential use against bacterial strains.

## 5 Conclusions

Sulfadimidine capped cobalt nanoparticles (Co-SD NPs) successfully synthesized by using sodium borohydride as wet-chemical reduction approach and sulfadimidine as capping agent. The as-synthesized Co-SD NPs are homogeneously dispersed, well distributed. The Co-SD NPs characterization was accomplished by using several characterization approaches. The XRD results clearly indicated that the crystalline phase is triclinic and average crystalline size is around 3.67 nm. The HR-TEM results have disclosed the particle size of cobalt nanoparticles is approximately 3–5 nm. The FT-IR analysis clearly indicated that sulfadimidine acting as a capping agent for cobalt nanoparticles. The as-synthesized Co-SD NPs were evaluated for their biological activities. Moreover, the antibacterial properties, the zone of inhibition of Co-SD NPs nanoparticles shown the inhibitory effect against all tested bacteria. This current study revealed that Sulfadimidine can be conjugated with Co-SD NPs to enhance the antibacterial activity against gram-negative bacteria. *In vitro* bactericidal inspections exposed that the sulfadimidine capped cobalt nanoparticles (Co-SD NPs) showed substantial activity with zone of inhibition of *Klebsiella pneumonia* 26 mm*, Escherichia coli* 22 mm*, and Pseudomonas syringae* 22 mm. The results obtained for *Klebsiella pneumonia* showed almost equal to that of the ampicillin antibiotic. Whereas, the antibacterial activity on *Escherichia coli* and sulfadimidine are same and comparatively less than reference antibiotic. Therefore, the dual nature of the sulfadimidine controls the size and also enables the cobalt nanoparticles stabilization. This expressively improves the penetration capability of the nanoparticles into the bacteria cell wall and thus outcomes in the enhanced biological activity of sulfadimidine capped cobalt nanoparticles.

## Declaration of Competing Interest

The authors declare that they have no competing interests.
